# Power spectral density-based resting-state EEG classification of first-episode psychosis

**DOI:** 10.1038/s41598-024-66110-0

**Published:** 2024-07-02

**Authors:** Sadi Md. Redwan, Md Palash Uddin, Anwaar Ulhaq, Muhammad Imran Sharif, Govind Krishnamoorthy

**Affiliations:** 1 Department of Computer Science and Engineering, University of Rajshahi, Rajshahi, 6205 Bangladesh; 2 Department of Computer Science and Engineering, Hajee Mohammad Danesh Science and Technology University, Dinajpur, 5200 Bangladesh; 3 School of Information Technology, Deakin University, Geelong, VIC 3220 Australia; 4 School of Engineering and Technology, Central Queensland University Australia, 400 Kent Street, Sydney, NSW 2000 Australia; 5 Department of Computer Science, Kansas State University, Manhattan, 66506 KS USA; 6 School of Psychology and Wellbeing, University of Southern Queensland, Ipswich, QLD Australia

**Keywords:** First-episode psychosis, EEG, PSD, GPC, Machine-learning, Computational biology and bioinformatics, Biomarkers, Diseases

## Abstract

Historically, the analysis of stimulus-dependent time–frequency patterns has been the cornerstone of most electroencephalography (EEG) studies. The abnormal oscillations in high-frequency waves associated with psychotic disorders during sensory and cognitive tasks have been studied many times. However, any significant dissimilarity in the resting-state low-frequency bands is yet to be established. Spectral analysis of the alpha and delta band waves shows the effectiveness of stimulus-independent EEG in identifying the abnormal activity patterns of pathological brains. A generalized model incorporating multiple frequency bands should be more efficient in associating potential EEG biomarkers with first-episode psychosis (FEP), leading to an accurate diagnosis. We explore multiple machine-learning methods, including random-forest, support vector machine, and Gaussian process classifier (GPC), to demonstrate the practicality of resting-state power spectral density (PSD) to distinguish patients of FEP from healthy controls. A comprehensive discussion of our preprocessing methods for PSD analysis and a detailed comparison of different models are included in this paper. The GPC model outperforms the other models with a specificity of 95.78% to show that PSD can be used as an effective feature extraction technique for analyzing and classifying resting-state EEG signals of psychiatric disorders.

## Introduction

Psychosis is a symptom commonly associated with an extended array of neurological and psychiatric disorders, including schizophrenia spectrum (schizophreniform, schizoaffective, and paranoid schizophrenia). The first episode of psychosis in schizophrenia can be hard to distinguish from other forms of psychosis. An early diagnosis relies heavily on identifying trait markers of schizophrenia in first-episode psychosis (FEP/first-episode schizophrenia/FESz) patients. Electroencephalography (EEG) has been tremendously successful in the time–frequency analysis of neural activation patterns during different cognitive and behavioral assessments. Recent resting-state studies show that EEG can also be used to decode intrinsic brain activity in a task-negative state. Multiple studies involving spectral analysis support the alterations in resting-state delta/alpha activity in the schizophrenia spectrum^1–3^. Although researchers have recently found significant spectral entropy modulation deficits with task performance in patients with FEP/FESz, they did not find any significant pre-stimulus spectral entropy differences. Current speculation is that it reflects a deficit in the synchronization of the neural assemblies that underlie cognitive activity^4^. On the other hand, several cortical alpha networks have been shown to be pathological in FEP patients in a recent resting-state magnetoencephalography (MEG) study^5^. Interestingly, Power Spectral Density (PSD) has been used in analyzing the alpha band default mode network (DMN) in schizophrenia in another MEG analysis^6^. This raises the question of whether PSD can also be used as a potential biomarker for EEG analysis to identify FEP patients accurately.

EEG is a waveform representation of the (electrical) brain signals measured by the fluctuations of voltage induced by the neuronal ionic activity^7^. The effectiveness of EEG in decoding neurological and emotional states of the brain is attributed to the high temporal resolution of the signal^8^ and our understanding of which frequency or pattern of the signal relates to a particular task, stimulus, or emotion. Several visual, auditory, and task-based stimuli have been developed over the years by researchers on account of EEG studies. These studies have eventually built the foundation of modern EEG-based emotion recognition, seizure detection, medical diagnosis, and brain-computer interface (BCI) systems. In particular, EEG is currently established as the primary method for seizure detection^9^. In contemporary EEG and MEG studies, delta, and alpha powers have been affiliated with attention and prolonged focus, signifying spontaneous resting-state brain activity. A more generalized model using multiple robust feature extraction techniques for highly accurate schizophrenia classification has also been proposed recently^10^. Several studies support the use of PSD as an effective EEG feature extraction method for machine-learning classification^11,12^. In another study, researchers used PSD of multiple frequency bands along with fuzzy entropy and functional connectivity for generalized anxiety disorder (GAD) classification with 97.83 (± 0.4)% accuracy^13^. This signifies the potential utility of combining the spectral features of multiple bands for the EEG classification of FEP. The core objective of this work is to combine the PSD of delta (0.5–4 Hz), theta (4–8 Hz), alpha (8–12 Hz), and low-beta (12–16 Hz) bands of resting-state EEG for the machine learning approaches. Since high-frequency gamma waves are typically associated with task or event-related potentials, only the low-to-medium frequency bands are chosen to investigate whether the EEG features associated with intrinsic brain activity have a significant difference that can be classified using machine learning. Another goal is to evaluate which machine learning models perform best for these features.

Machine learning models for EEG classification have been popularized with the success of linear discriminant analysis (LDA), support vector machine (SVM), and neural networks in multiple EEG paradigms. A random forest classifier has been proposed for the classification and analysis of mental states using single-channel EEG^14^. SVM has been successfully used in multiple sclerosis^15^ and epilepsy detection^16^. Gaussian Process Classifier (GPC) has also been proposed for classifying mental states^17^ and detecting neonatal seizures^18^. In this work, we analyze the effectiveness of multiple methods, namely random forest, SVM, and GPC, for classifying FEP patients and healthy controls based on the PSD of multiple EEG frequency bands. A medium-sized dataset of 28 controls and 44 patients has been balanced using borderline-SMOTE^19^ for this work. With a very small number of parameters, the computationally efficient GPC has performed very well, with an accuracy of 95.51 (± 1.74)% and a specificity of 95.78 (± 3.3)%. The dataset used in this work is associated with the MEG study by Salisbury et al*.* in which machine learning network analysis of resting alpha-band neural activity identified several aberrant networks in FEP including the left temporal, right inferior frontal, right posterior parietal, and bilateral cingulate cortices^5^.

*Contribution*: The present study demonstrates a distinct and novel contribution in the field by leveraging the combined power spectrum of multiple frequency bands in resting-state EEG to detect FEP, offering promising clinical applications. This innovative framework establishes a fundamental groundwork for accurately classifying FEP and control subjects using resting-state EEG data. We anticipate that future advancements will build upon this foundation, employing more sophisticated neural network models and integrating various feature extraction techniques based on time–frequency analysis to enhance classification performance and diagnostic accuracy further. The potential for continued refinement and expansion of this framework underscores its significance in FEP detection.

## Materials and methods

### Electroencephalography (EEG)

Most publicly available EEG datasets are focused on diverse neural activation events of healthy and occasionally pathological brains. That being said, the publication of resting-state EEG studies and datasets has also increased in the past few years. Major depressive disorder^20^, depression^21,22^, cognitive states^23^, and multiple other psychiatric disorders^22^ have been studied using resting-state EEG as of late, and some of them have been published as datasets. In addition to the MEG study of resting-state cortical alpha networks of FEP/FESz^5^, Salisbury et al*.* also published the corresponding EEG datasets in 2022^24,25^. To obtain resting data, EEG was recorded for 5 min using an Elekta Neuromag Vectorview system with a low-impedance 60-channel cap following the 10-10 system. For our work, we use the *Resting Task 1* dataset, excluding the *Resting Task 2* samples of 10 subjects that are also present in the *Resting Task 1* dataset. The subject population consists of 72 subjects (44 patients and 28 controls matched for age, gender, and estimated premorbid IQ). In particular, MEG data was recorded for 53 participants while EEG data was collected for 72 participants. The MEG and EEG datasets are separate and only the EEG datasets were publicly available. The phenotype directory contains clinical assessment results and data, organized by type, for all subjects. The assessment results are categorized as follows: BPRS: Brief Psychiatric Rating Scale, SANS: Scale for the Assessment of Negative Symptoms, SAPS: Scale for the Assessment of Positive Symptoms, GAFGAS: Global Assessment of Functioning, SFS: Social Functioning Scale MATRICS: MATRICS Consensus Cognitive Battery, WASI: Wechsler Abbreviated Scale of Intelligence, and Hollingshead: Hollingshead Four-Factor Index of Socioeconomic Status. For the medications information is given as follows: Chlorpromazine equivalency of prescribed medication at the time of the EEG scan. The demographic information of the subjects is presented in Table 1.

**Table 1:** Demographic information of the subject population.

Group	N (male, female)	Average age (SD)	Ethnicity—White, Black, Asian, mixed, undisclosed
All subjects	72 (46, 26)	21.96 (4.66)	46, 17, 5, 3, 1
Control	28 (16, 12)	21.33 (3.88)	21, 4, 3, 0, 0
FEP	44 (30, 14)	22.36 (5.06)	25, 13, 2, 3, 1

The dataset is obtained from OpenNeuro^26^ (accession number: ds003944). It is available under the Creative Commons License (CC0). The phenotypic information is also included in the dataset. The cognitive and socio-economic assessments have been conducted using the MATRICS score and SES score respectively, and the negative effects of FEP are evident in the patient population.

### Preprocessing

The initial step of every EEG study is preprocessing the data to reduce the effects of several unwanted artifacts. The EEG signals used in this work are obtained in a 5-min period using a low-impedance 10-10 system 60-channel cap (Elekta Neuromag Vectorview system), with a sampling frequency of 1000 Hz. The online reference used in this system is the linked mastoids. Two additional electrooculogram (EOG) channels and an electrocardiogram (ECG) channel are also included in the data. EOG channels are particularly important as they capture the eye-blink artifacts that are also present in the EEG signals. Much work has been done to establish a correct method for EOG-related artifact removal based on Independent Component Analysis (ICA) and regression^27^. EEG signals also correlate with the ECG signal (heartbeat artifacts), which can be removed using ICA^28^ and Signal-Space Projection (SSP).

**Figure 1:**
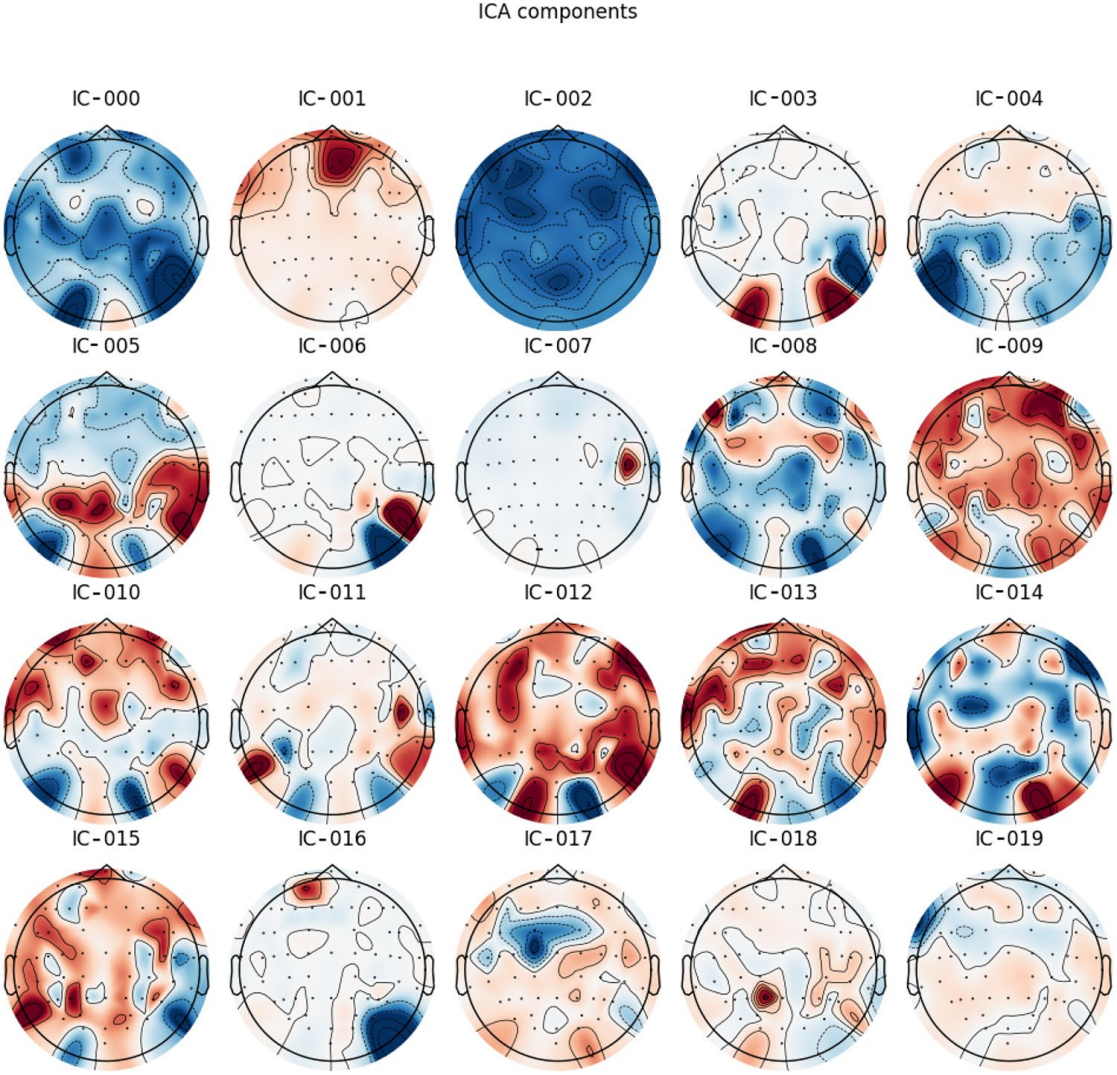
All 20 ICs for a subject. From a cursory glance, the IC-001 and IC-002 appear to be related to unwanted artifacts. IC-001 is close to the eyes, which indicates EOG-related potential, and IC-002 appears to be incoherent compared to the other ICs.

ICA is a blind source separation (BSS) technique that has revolutionized signal separation from mixed signals and has been used in numerous EEG and fMRI studies over the years. With the success of a fast and efficient ICA implementation, fittingly named FastICA^29^, it has become much easier to remove artifacts from EEG signals. In this work, FastICA is used to remove both EOG and ECG artifacts separately. We apply temporal band-pass filtering of 0.5–35 Hz before applying ICA to remove low-frequency drifts and high-frequency components that are not needed for this study. We extract 20 Independent Components (ICs) from all the channels to find out which components correspond to EOG and ECG artifacts and remove those components. The ICs for a sample subject are shown in Fig. 1.

**Figure 2:**
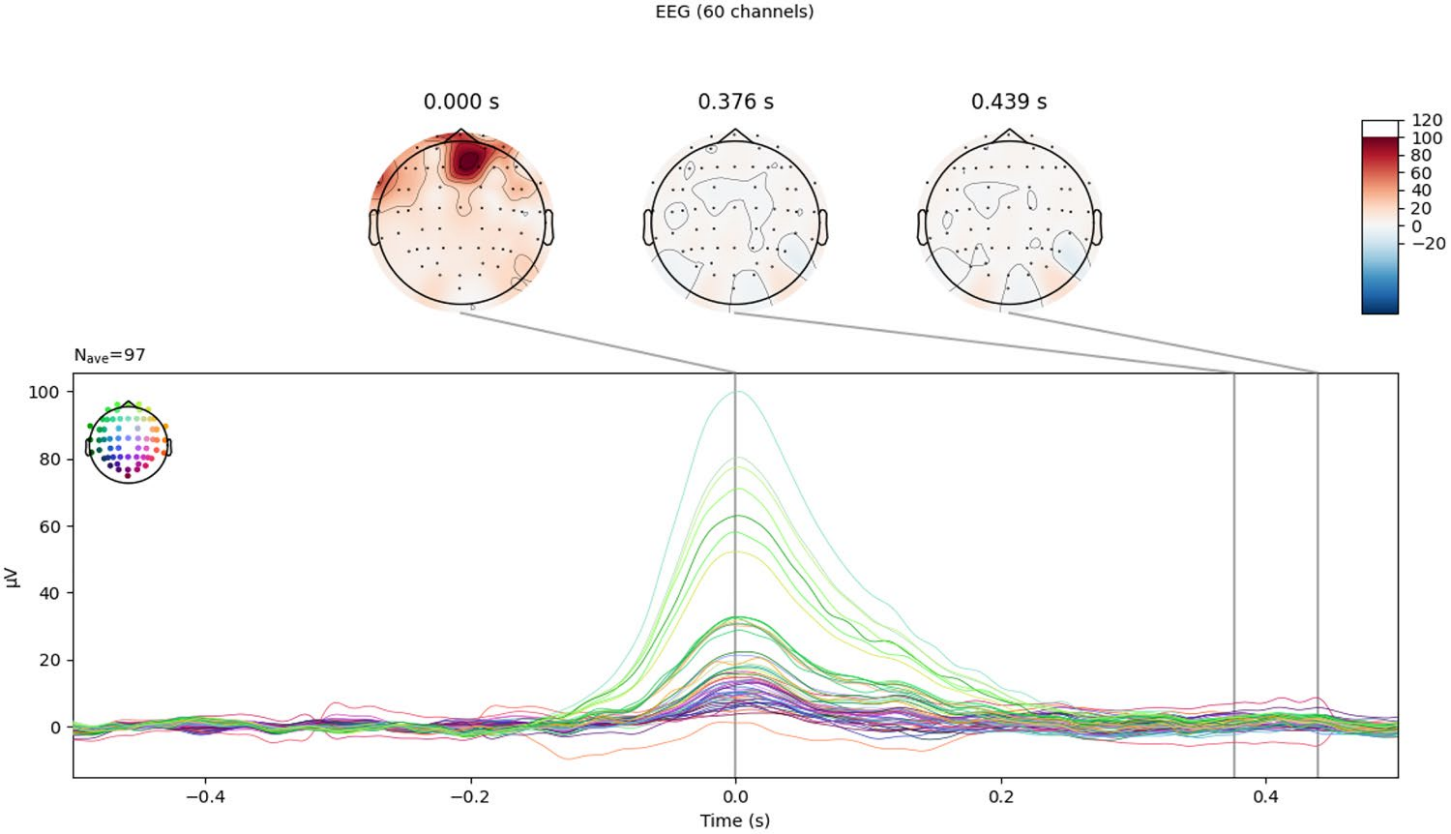
The ICs identified to be EOG-related IC (− 0.5 to 0.5 s range, 1000 time points).

A correlation method is used to detect EOG-related ICs in EEG data, based on the Pearson correlation between the filtered data and the filtered EOG channels. The thresholding is performed using an adaptive z-scoring approach, where components with z-scores above the threshold are flagged and masked. This process is repeated iteratively until no supra-threshold component remains, as shown in Fig. 2.

**Figure 3:**
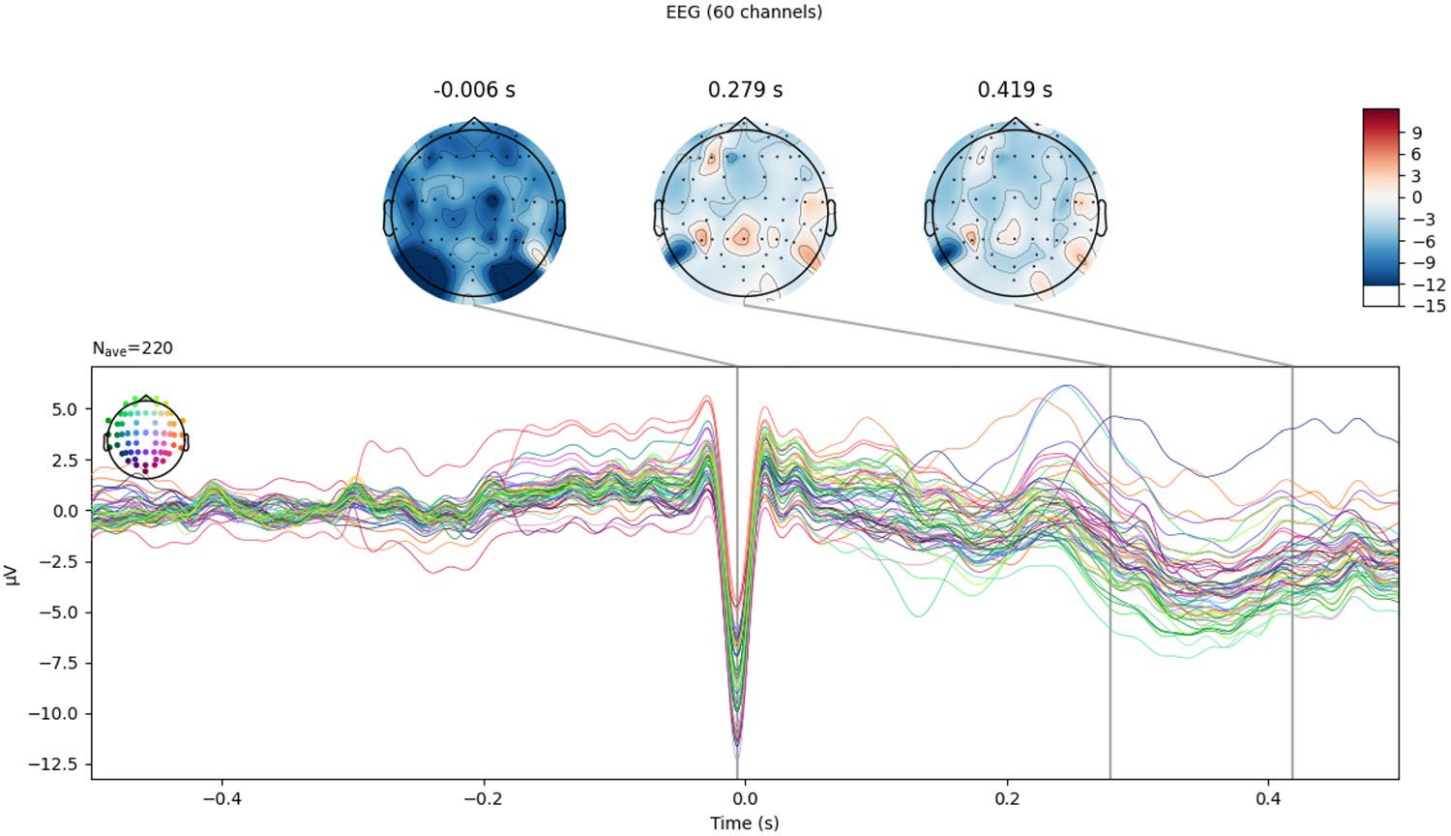
IC(s) identified to be ECG-related IC (− 0.5 to 0.5 s range, 1000 time points).

**Figure 4:**
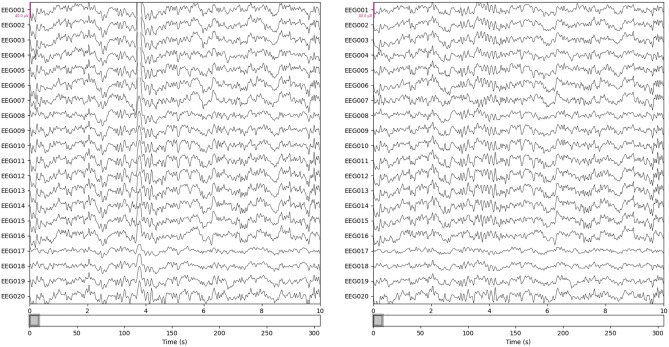
Effect of artifact removal. The original signals are shown in the left panel, and the processed signals are in the right panel. 20 out of 60 channels are shown with 0.5–16 Hz bandpass filtering in a 10 s window; EOG artifacts are visible at ~ 4 s timestamp in the left panel.

The ECG-related ICs are also identified using the same principle. Phase statistics^30^ are also applied to identify the heartbeat artifacts since these artifacts do not affect each EEG electrode with the same potential due to the temporal properties of the ECG signal. Figure 3 shows the ICs that correlate to the ECG signal, and Fig. 4 shows the effect of EOG and ECG-related artifact removal.

### Cross-spectral density (CSD)

Before proceeding to the feature extraction step, we verify sensor-to-sensor coherence by calculating the CSD of the channels to justify using spectral features for further analysis. The key objective of CSD is to compare two signals by measuring the spectral power distribution and determining the coherence between them. This analysis helps in understanding the relationship between the signals in the frequency domain, which is crucial for ensuring that the sensors are recording coherent data that can be meaningfully analyzed together. CSD can be achieved through various methods, such as the Morlet wavelet (Continuous Wavelet Transform/wavelet decomposition) and Short-Time Fourier Transform (STFT). These methods decompose signals into their time–frequency components, allowing for a detailed spectral analysis. In our approach, we utilize the Morlet wavelet to decompose every signal into time–frequency components, which facilitates the calculation of the spectral correlation of the signals. For each frequency band, we specify eight equidistant values (frequency scales) ranging from the lower-bound to the upper-bound. This detailed decomposition enables us to capture the coherence across different frequency scales, ensuring robust spectral feature extraction for subsequent analysis. The wavelet power spectrum can be defined as

1 $$\left( {WPS} \right)_{x} \left( {\tau ,s} \right) = {}W_{x} \left( {\tau ,s} \right){}^{2} ,$$

where *W*_*x*_ is the wavelet transform and *τ*, *s* represent the position of the wavelet in the time and frequency domain, respectively^31^. The Morlet wavelet is given by

2 $$\psi \left( x \right) = exp\left( { - \frac{{x^{2} }}{2}} \right)cos\left( {5x} \right).$$

**Figure 5:**
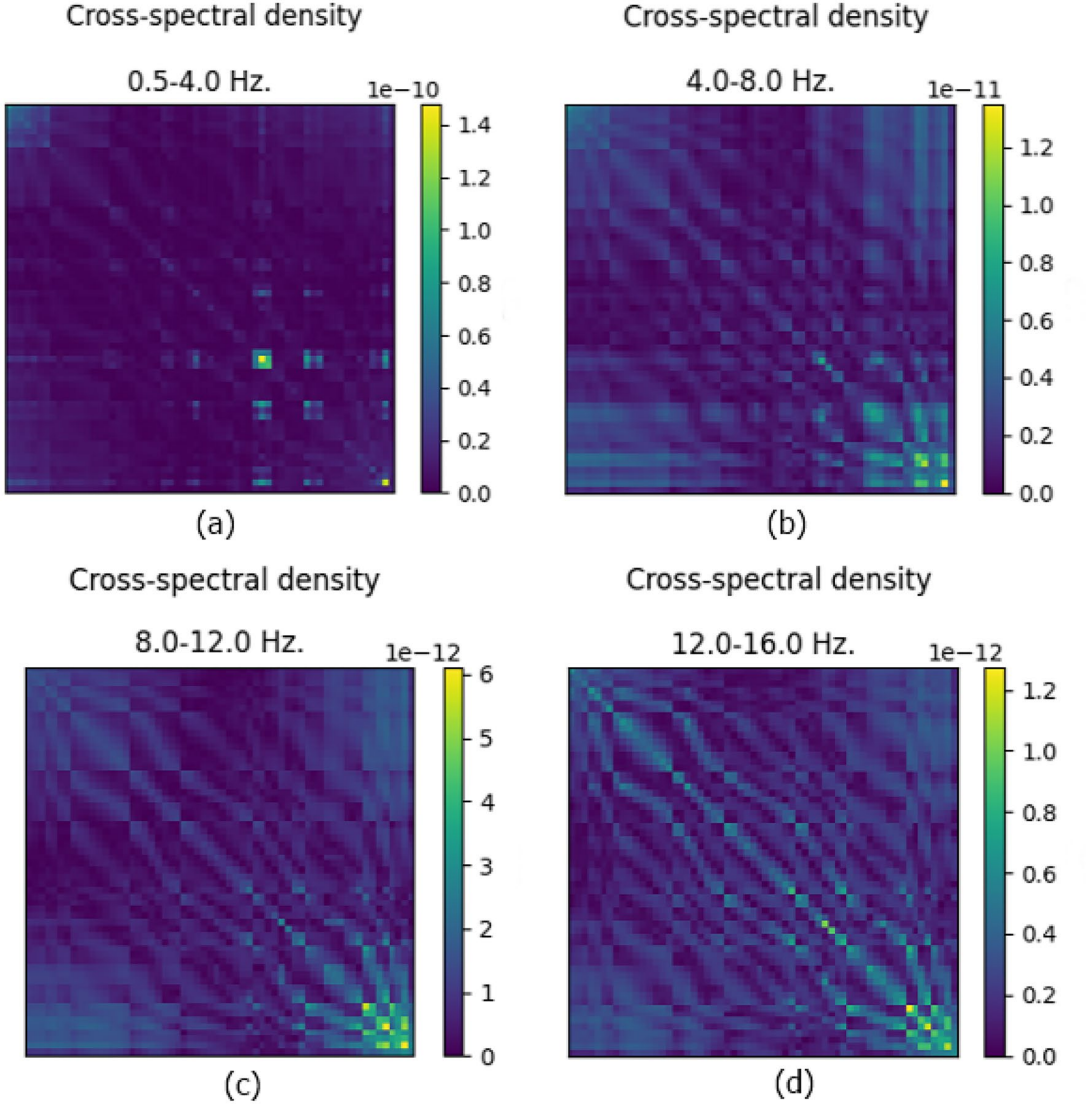
CSD analysis of a single subject. (**a**) delta, (**b**) theta, (**c**) alpha, and (**d**) low-beta CSD matrices denote coherence across channel signals.

By combining the correlation between the power spectrums for each pair of signals, we eventually get a 60 × 60 matrix for all 60 channels. The average CSD matrices for a sample subject across different frequency bands are presented in Fig. 5.

### Power spectral density (PSD)

**Figure 6:**
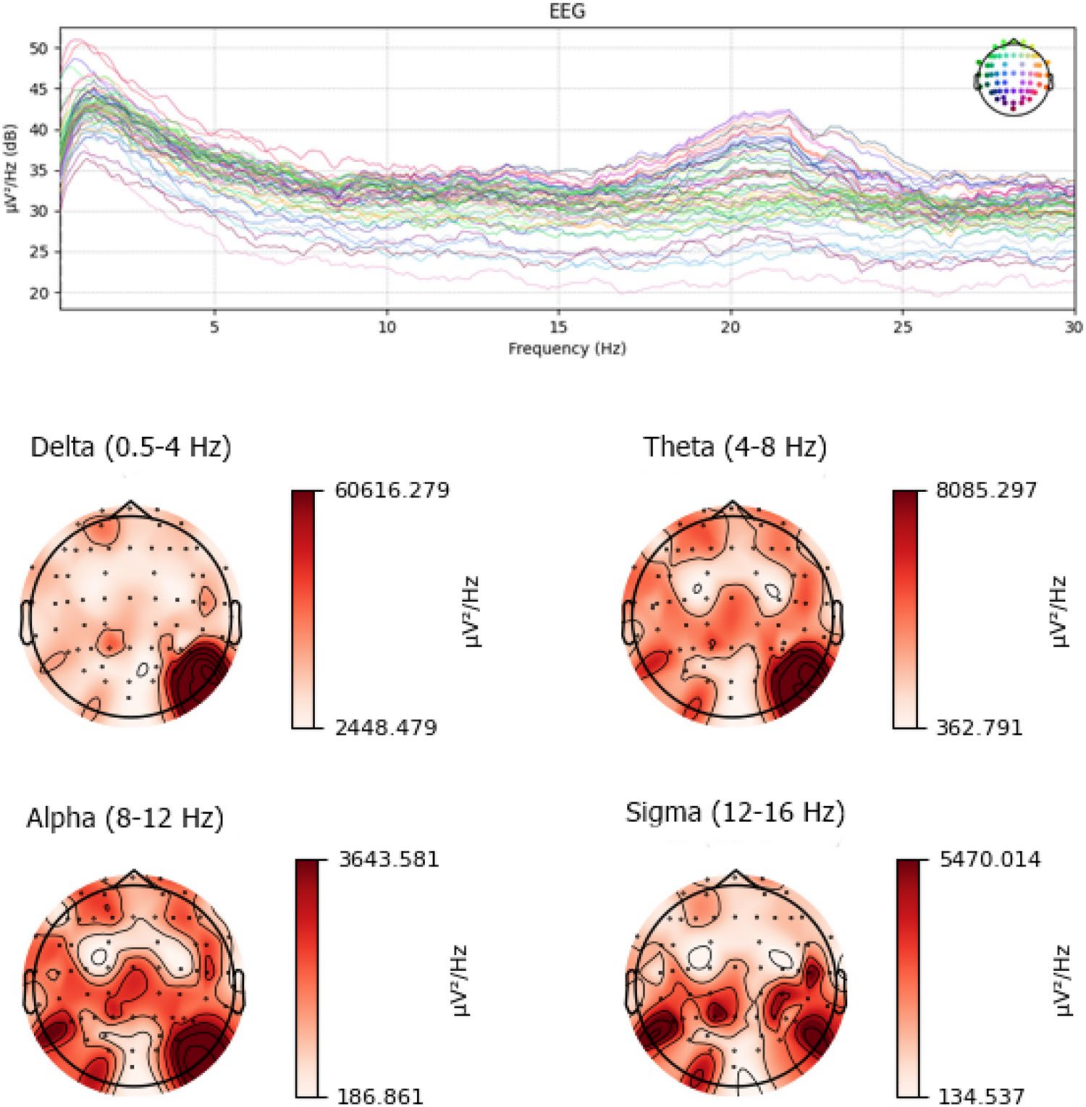
Power spectral representation of EEG data. Each frequency band shows the characteristic PSD of the signal.

PSD is an effective method to differentiate between noise and features in a signal by making a spectral representation of the power distribution of its frequency components. Thomson’s multitaper spectral estimation method is used to compute PSD in this work^32^. The multitaper method is preferred over other similarly popular methods such as Welch’s method and simple FFT for computing PSD because it results in lower variance, increased frequency resolution, and reduced bias. This method starts by calculating a periodogram for each of the first *K*≈2NW Discrete Prolate Spheroidal Sequences (DPSS/Slepian tapers)^33^ and then averaging these periodograms. Figure 6 shows the power spectra of a sample subject’s preprocessed EEG data in μV^2^/Hz (decibels).

Resting state EEG signals are typically characterized by low amplitude and frequency fluctuations, which can make it difficult to distinguish between different states or conditions. By dividing the data into smaller segments, the EEG signals can be assumed to be more stationary within each segment, allowing for more robust analysis and classification. It is important to mention that, even though the CSD matrix is used to verify sensor-to-sensor coherence in this work, the main focus is the combined PSD of all the channels and not the sensor locations for the features. In summary, the data is divided into 30 s segments, and four PSD bands for each subject are computed. The four bands are then combined for the classification step. Only the PSD features are used for machine learning classification without further processing or dimensionality reduction in this work. Segmenting EEG data of ~ 5 min length into 30 s results in 9 to 10 segments for each subject. The four frequency bands combined produce 240 PSD features for each segment which is adequate for machine learning classification.

### Random forest

Random forest is a tree-based ensemble learning technique^34^ that has been used many times in different classification tasks. The core idea of a random forest classifier is to combine multiple decision trees using an ensemble (bagging) mechanism. The prediction of the random forest is given by the averaged prediction of the decision trees combined with the extremely randomized method^35^. A random forest of 200 decision trees with a maximum depth of 30 per tree is used in this work to classify PSD feature vectors.

### Gaussian process classifier (GPC)

The GPC for binary classification is based on Laplace approximation^36^. With the joint probability $p\left( y \right)p\left( {x{|}y} \right)$ derived from Bayes’ theorem, where *y* denotes the class label, the marginal likelihood *p*(*y*|*X*) is given by

3 $$p\left( {y|X} \right) = \smallint p\left( {y|f} \right)p\left( {f|X} \right)df = \smallint exp\left( {\Psi \left( f \right)} \right)df.$$

Using a Taylor expansion of Ψ(*f*) the approximation *q*(*y*|*X*) to the marginal likelihood is derived as follows.

4 $$p\left( {y|X} \right) \simeq q\left( {y|X} \right) = exp\left( {\Psi \left( {\hat{f}} \right)} \right)\smallint exp\left( { - \frac{1}{2}\left( {f - \hat{f}} \right)^{T} A\left( {f - \hat{f}} \right)} \right)df.$$

An approximation to the log marginal likelihood is derived by analyzing this Gaussian integral.

5 $$log\,q\left( {y|X,\theta } \right) = - \frac{1}{2}\hat{f}^{T} K^{ - 1} \hat{f} + log\,p\left( {y|\hat{f}} \right) - \frac{1}{2}log|B|,$$

where

6 $$|B| = |K| \cdot \left| {K^{ - 1} + W} \right| = \left| {I_{n} + W^{\frac{1}{2}} KW^{\frac{1}{2}} } \right|,$$

and *θ* is a vector of hyperparameters of the covariance function.

We use a stationary covariance function or radial basis function (RBF), as the Gaussian process kernel. With $r = x - x_{i}$ and a specified shape parameter *ε*, the Gaussian RBF is given as follows.

7 $$\varphi \left( r \right) = exp\left( { - \left( {\varepsilon r} \right)^{2} } \right)$$

### Support vector machine (SVM)

Support vector machines (SVMs) are widely used for classification because they build a linear decision surface from a very large feature space to which input vectors are mapped non-linearly^37^. Based on the properties of the optimal hyperplane (feature map), the SVM algorithm can be classified into linearly separable, linearly inseparable, and non-linearly separable. For non-linear feature mapping, a kernel function is used to map the inputs implicitly. Similar to the GPC, we use the Gaussian RBF as the kernel function for our SVM model. For Gaussian RBF, *φ* the kernel function can be written as

8 $$K\left( {x_{i} ,x_{j} } \right) = \varphi \left( {x_{i} } \right) \cdot \varphi \left( {x_{j} } \right).$$

Then the vector to the hyperplane (weight) is given by

9 $$w = \mathop \sum \limits_{i} \upalpha _{i} y_{i} {\varphi }\left( {x_{i} } \right)$$

The SVM classifier minimizes the following expression to separate the input feature vectors with the parameter *λ* > 0, which denotes the tradeoff between the size and flexibility of the margin for classification.

10 $$\left[ {\frac{1}{n}\mathop \sum \limits_{i = 1}^{n} \max \left( {0,1 - y_{i} \left( {w^{T} x_{i} - b} \right)} \right)} \right] + \lambda |w|^{2}$$

### Multilayer perceptron (MLP) model

A multilayer perceptron (MLP) model was employed to classify the EEG data. The MLP is a type of artificial neural network consisting of multiple layers of neurons: an input layer, one or more hidden layers, and an output layer. Each neuron in a layer is connected to every neuron in the subsequent layer, and the model uses a non-linear activation function to capture complex patterns in the data. For this study, we optimized the number of hidden layers, the number of neurons per layer, the activation functions, and other hyperparameters using cross-validation to achieve the best classification performance. MLPs have been widely used in EEG signal classification due to their ability to model complex, non-linear relationships in the data.

### AdaBoost classifier

An AdaBoost classifier was also utilized in this study. AdaBoost, or Adaptive Boosting, is an ensemble learning technique that combines multiple weak classifiers to form a strong classifier. The algorithm iteratively trains weak classifiers, typically decision stumps, by focusing on the samples that were previously misclassified. This process is repeated, with each classifier's predictions weighted based on their accuracy, to improve overall model performance. We tuned the number of estimators and learning rate to find the optimal configuration for classifying the EEG data. AdaBoost has proven effective in various classification tasks, including EEG signal classification, due to its ability to enhance the performance of weak learners.

### Low resolution electromagnetic tomography (LORETA) analysis

LORETA analysis estimates the sources of brain activity from EEG signals by addressing the inverse problem using a smoothness constraint. The process involves preprocessing EEG data, accurately positioning electrodes, defining a 3D brain grid, calculating a lead field matrix, and applying the LORETA algorithm to estimate and visualize source activity. It offers a non-invasive and cost-effective method with high temporal resolution, although it has lower spatial resolution compared to fMRI. LORETA is widely used in cognitive neuroscience and clinical research to study brain function and disorders, despite the inherent ambiguity of the inverse problem.

## Results

**Figure 7:**
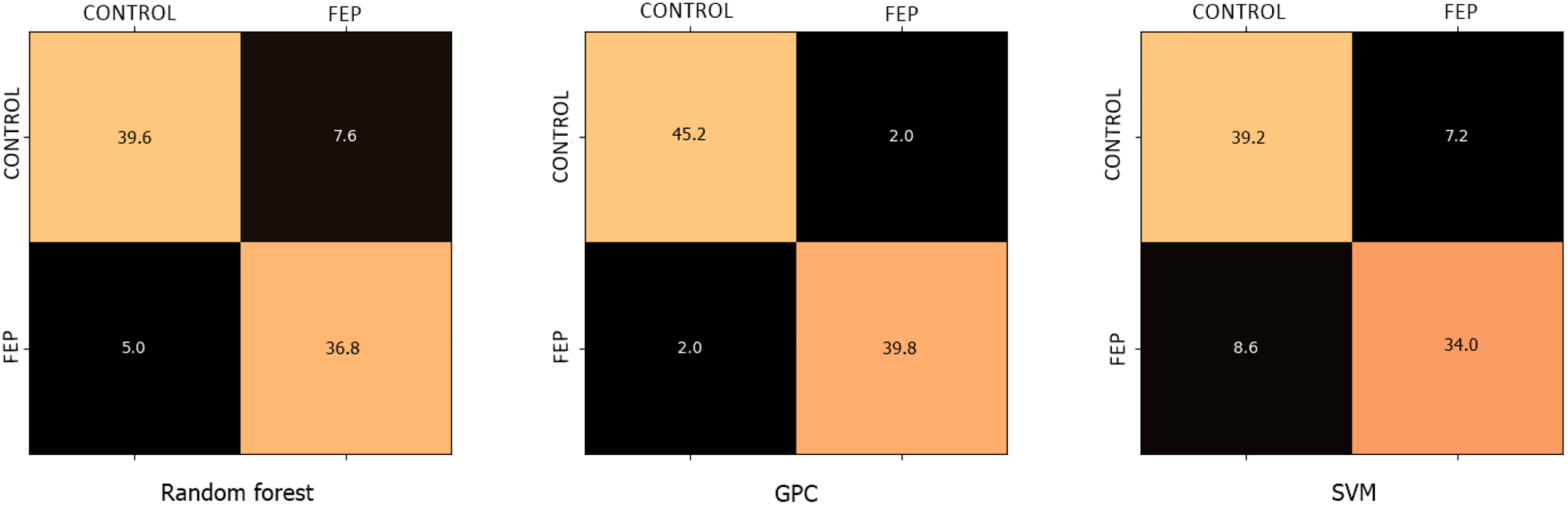
Confusion matrices averaged across test data.

The experiments were done using MATLAB R2022b and Python 3.10 in Microsoft Windows 11 (22H2) platform on an AMD Ryzen 7 3750H computer. The performance of each model is evaluated using fivefold cross-validation with 80% data used for training and 20% for testing. Note that using a larger training set ratio for EEG classification is crucial for model accuracy and generalization^38^. It allows the model to learn complex patterns, avoid overfitting, and achieve robust parameter estimation. Additionally, it increases the statistical power of the model, essential for detecting subtle differences in clinical settings. As such, this approach supports rigorous validation, enhancing model robustness and reliability, which is vital for clinical applications like diagnosing first-episode psychosis. Consequently, we set the training-to-testing ratio as 80% to 20%. The final confusion matrix for each model is derived by taking the average of all confusion matrices, as shown in Fig. 7.

Initially, we applied principal component analysis (PCA) for feature reduction. However, PSD features from the higher frequency bands exhibited dominant variance. Surprisingly, while PCA aimed to streamline features, it inadvertently led to the elimination of lower-frequency features, contrary to our expectations. This unexpected outcome prompted us to explore diverse model parameters and kernels. We found that retaining all 240 features produced optimal results for certain models without succumbing to overfitting. It is important to note that PCA requires around 1000 cases for reliable analysis when using 240 features. Consequently, we proposed our PSD-grounded approach for feature reduction, detailed in our methodology, based on empirical evidence to ensure the robustness of our findings.

We use precision, recall, and F1-score to evaluate the classification accuracy for each class. The mathematical expressions for precision, recall, and F1-score are as follows.

11 $$Precision = \frac{TP}{{TP + FP}},$$

12 $$Recall = \frac{TP}{{TP + FN}},$$

13 $$F1 = \frac{2 \times Precision \times Recall}{{Precision + Recall}},$$

where *TP*, *FP*, and *FN* denote true-positive, false-positive, and false-negative predictions respectively. Specificity or true negative rate is defined as the recall of the negative class (control). The accuracy score, precision, recall, and F1 scores for the random forest, GPC, and SVM models are discussed in Tables 2, 3, and 4, respectively.

**Table 2:** Classification report for the random forest model.

Group	Precision (SD)	Recall (SD)	F1-score (SD)	Overall accuracy (SD)
Control	89.2 (± 4.8)	83.93 (± 1.9)	86.34 (± 2.3)	85.84 (± 2.72)
FEP	82.89 (± 1.9)	89.2 (± 4.9)	85.27 (± 3.2)

**Table 3:** Classification report for the GPC model.

Group	Precision (SD)	Recall (SD)	F1-score (SD)	Overall Accuracy (SD)
Control	**95.93** (± 3.5)	**95.78** (± 3.3)	**95.72** (± 1.7)	**95.51 (± 1.74)**
FEP	**95.56** (± 3.5)	**95.3** (± 3.1)	**95.26** (± 1.8)	

Significant values are in bold.

**Table 4:** Classification report for the SVM model.

Group	Precision (SD)	Recall (SD)	F1-score (SD)	Overall accuracy (SD)
Control	82.49 (± 3.6)	84.69 (± 4.2)	83.45 (± 2.2)	82.25 (± 2.18)
FEP	82.47 (± 3.4)	79.45 (± 5.3)	80.75 (2.5)	

In addition to these models, a multilayer perceptron model and an AdaBoost classifier were employed that achieved 59.65% and 61.41% accuracy respectively. With an accuracy of 95.51 (± 1.74)% and specificity of 95.78 (± 3.3)%, the GPC model has outperformed the other models (↑9.67% accuracy over random forest and ↑13.26% accuracy over SVM) and thus, decided as the best model for PSD-based classification of FEP vs. control. The proposed GPC model has a comparatively small number of parameters and can be considered a ‘shallow’ learning model. The high accuracy of GPC can be attributed to selecting a suitable covariance function for the input features. Other RBF kernels should also be considered for comparison. Deep recurrent neural network (RNN) models trained with time–frequency features, much like the recently proposed models for epilepsy classification, age prediction, and concussion classification^39–41^, can hypothetically outperform this model. Another aspect that requires further analysis is the method for computing PSD. Future studies should also consider Welch's method for computing PSD to compare with the results of the DPSS method.

## Discussion

**Figure 8:**
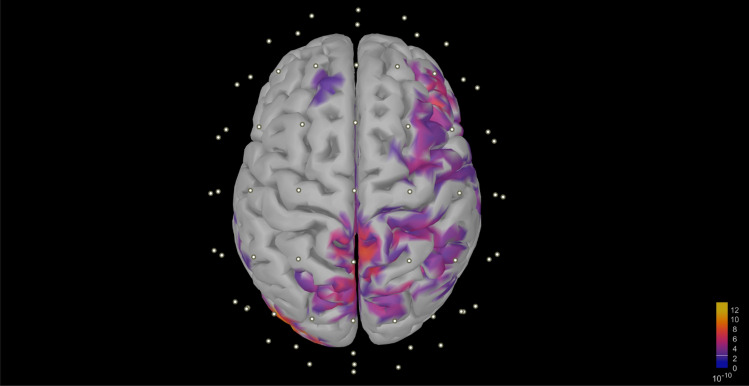
Contrast in cortical EEG source powers between control and FEP individuals using sLORETA source estimation (p < 0.05).

In order to elucidate the underlying neurophysiological basis for the classification results, we employed sLORETA to estimate cortical EEG sources for the weighted grand average signal of the control and FEP groups^42^. Subsequently, a t-test was conducted to compare the power spectral differences between the cortical source estimates of the two averaged signals. The results revealed significant disparities in source powers (Fig. 8), which align with the inferences derived from the machine learning models. This finding suggests that the classification success achieved by the algorithms can be attributed to the distinctive cortical EEG source powers in control and FEP individuals. These results provide further support for the potential utility of EEG-based classification methods in differentiating individuals with FEP from healthy controls.

In the MEG study associated with the data used in this work, four cortical alpha networks are described as pathological in individuals with first-episode psychosis. These networks involve the bilateral anterior and posterior cingulate; left auditory, medial temporal, and cingulate cortex; right inferior frontal gyrus and widespread areas; and right posterior parietal cortex and widespread areas. Since individual anatomical data were not available, we used the standard MNI152 brain template for EEG source estimation in the sample subjects. We hypothesize that accurate source estimation based on individual subject anatomy (structural MRI), combined with machine learning, can provide insights similar to those of the MEG study. In summary, resting-state PSD is shown to be an appropriate feature in the EEG-based analysis of FEP, and the proposed GPC model emerges as the best model for such features, achieving the primary goal of this study as outlined. Future work should focus on EEG sources as well as combined PSD for machine learning-based inferences.

## Conclusion

In this study, we have evaluated the use of machine learning methods for the classification of patients with first-episode psychosis (FEP) and healthy controls based on the Power Spectral Density (PSD) of resting-state EEG. We have reviewed various feature engineering techniques and machine learning models to demonstrate that FEP patients can be accurately detected utilizing resting-state EEG. In addition, we have demonstrated that low-to-medium frequency (delta-to-low-beta band) waves are pathological in FEP patients and can differentiate patients from healthy persons with the same degree of accuracy as task/event-related high-frequency waves. PSD is shown to be a reliable characteristic for the effective classification of FEP using machine learning. We conclude that resting-state EEG studies can lead to an accurate diagnosis of FEP/FESz and other psychiatric disorders and should be regarded as equally essential as stimulus-based EEG studies. As this study focuses solely on developing a machine learning model using PSD-based features for the resting-state EEG classification of first-episode psychosis, comparing it with purely statistical models such as ANOVA could be explored in future. In addition, understanding the pathological state could be a valuable area for future research.

## Data Availability

The denoised and preprocessed data used in this work is available at https://zenodo.org/record/7315010 while the original *EEG: First Episode Psychosis vs. Control Resting Task 1* dataset is available at 10.18112/openneuro.ds003944.v1.0.1.
